# Patient Experiences With Temporomandibular Disorders: A Survey on the Pathways in Diagnosis and Treatment

**DOI:** 10.1111/joor.13993

**Published:** 2025-05-14

**Authors:** Aurora Manfredini, Ovidiu Ionut Saracutu, Charles S. Greene, Marco Ferrari, Daniele Manfredini

**Affiliations:** ^1^ School of Dentistry University of Ferrara Ferrara Italy; ^2^ Department of Medical Biotechnologies University of Siena Siena Italy; ^3^ Department of Orthodontics University of Illinois at Chicago College of Dentistry Chicago Illinois USA

**Keywords:** biopsychosocial model, dental education, evidence‐based dentistry, patients' experiences, temporomandibular disorders, TMD specialist, TMD treatment

## Abstract

**Background:**

Despite the availability of evidence‐based guidelines on temporomandibular disorders (TMD), some past theories of TMD aetiology, diagnosis and treatment are still diffused among clinical practitioners.

**Objective(s):**

The study aims to gather and analyse patient experiences and outcomes related to their seeking care for TMD symptoms.

**Methods:**

In November 2023, a survey to collect comprehensive data on patients' diagnostic journeys, treatment experiences, and outcomes was distributed through online platforms and TMD patient support groups in a variety of countries. Correlation between different variables was assessed and comparisons were made between patients with a different history of treatment‐seeking experiences.

**Results:**

A total of 153 participants filled out the survey. Of them, 31.4% (*N* = 48) needed to consult two or three professionals, while some of them saw up to four or five (*N* = 27, 17.6%). Moreover, patients experienced a wide range of pain duration time before receiving a diagnosis. Almost one‐third of patients were in pain for more than one year (*N* = 43, 28.1%), and a remarkable number never received appropriate care (*N* = 30, 19.6%). Additionally, those patients who saw one or more dentists who emphasised abnormalities of dental occlusion or jaw position ended up consulting significantly more health providers before receiving a diagnosis than those who did not have that experience (*p* = 0.0062).

**Conclusion:**

There is a need to better implement knowledge about TMDs among health‐care providers, starting from improving the quality of education at the university course level and continuing into various post‐graduate educational offerings.

## Graphical abstract

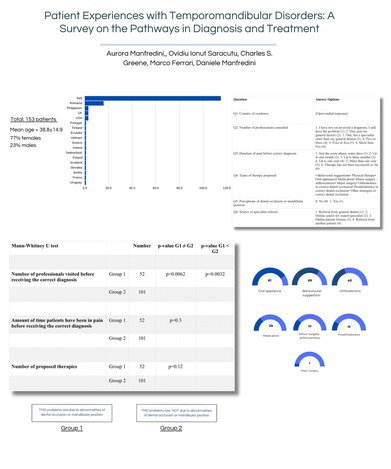

## 1 Introduction

Temporomandibular disorders (TMDs) is a collective term that is used to describe musculo‐skeletal disorders arising from the stomatognathic structures, including the masticatory muscles, the temporomandibular joints (TMJs) and associated structures [1]. These disorders may present with pain localised in the pre‐auricular area and/or in the masticatory muscles, jaw motion abnormalities and limitations, and articular sounds such as click and/or crepitus during mandibular movements [2]. TMDs can significantly affect an individual's quality of life through chronic pain and psychosocial dysfunction [3]. The prevalence of TMD symptoms ranges from 6% to 93% in the general population level, whilst only 3.6% to 7% of individuals have been estimated to require treatment during their lifetime [4, 5, 6, 7]. The majority of patients in clinical samples are females, likely due to the influence of biological and hormonal factors [8, 9, 10, 11, 12].

The aetiology of most TMD cases is multifactorial and complex, involving a combination of physical (axis I) and psychosocial (axis II) factors that play a critical role in contributing to the suffering, pain behaviour and disability associated with the patient's pain experience [1]. Currently, the most evidence‐based approach in the assessment and management of the cardinal features of TMDs is the biopsychosocial model [13, 14, 15, 16]. This is a comprehensive and interdisciplinary approach, starting with conservative treatments and reversible measures as the first line of therapy (e.g., counselling; physiotherapy; cognitive‐behavioural approaches; oral appliances without predetermined or forced occlusal designs; and pharmacotherapy for pain relief and muscle relaxation) [17]. In cases where conservative treatments fail, more invasive procedures must be considered [18], but many chronic pain cases will persist despite the best available care.

Despite the availability of evidence‐based guidelines, it seems that some misconceptions that characterise past theories of TMD aetiology, diagnosis and treatment are still widely believed by a significant number of clinical practitioners [19]. As discussed in some recent papers by Greene and Manfredini et al., some professionals continue to propose treatments that lack scientific support or are even potentially harmful: occlusal treatments, extensive prosthetic work, long‐term use of oral appliances, jaw repositioning and orthodontics [20, 21]. It is particularly important to remind that TMD treatment theories based on the correction of dental occlusion have been repeatedly undermined by a large number of experimental and clinical studies. For example, artificially induced occlusal interferences have been shown to have low clinical relevance and transient effects on healthy subjects [17]. Furthermore, there have been several investigations showing the neutrality of orthodontic treatment on the TMJs as well as systematic reviews suggesting that occlusal treatment is not useful for the treatment or prevention of TMDs [22]. This constantly growing body of scientific evidence underscores the need for continued education and adherence to evidence‐based practices in the management of TMD, with dentists embracing the role of caregivers [23].

The aim of this study is to investigate patients' experiences with TMD diagnosis and treatments. By analysing patient‐reported outcomes and diagnostic journeys, this research aims to identify areas where clinical practice may diverge from evidence‐based recommendations. In doing so, we will try to present a picture of the current practices that are commonly occurring within the dental practitioner community, and to discuss some potential patterns for more effective practices. Ultimately, this research seeks to inform clinicians and improve patient care by discussing how TMD treatments can be aligned with the best available evidence.

## 2 Materials and Methods

Data collection for this cross‐sectional study was conducted during the month of November 2023, by distributing a survey through online platforms and TMD patient support groups, targeting a broad and diverse demographic group in a variety of European countries. Participation was voluntary, and anonymity was strictly maintained to encourage open and honest responses. Data were used for research purposes only. Research was approved by the School of Dentistry, University of Siena (IRB #2023‐0137).

The survey provided the collection of data regarding their diagnostic journeys, treatment experiences and outcomes. The questionnaire consisted of six key questions (Table 1). Data from Q2, Q3 and Q5 were transformed into ordinal values, to be used for the statistical analysis.

**TABLE 1:** Key questions.

Question	Answer options
Q1. Country of residence	[Open‐ended response]
Q2. Number of professionals consulted	1. I have not yet received a diagnosis, I still have the problem. 2. One, just my general dentist. 3. One, but a specialist other than my general dentist. 4. Two or three. 5. Four or five. 6. More than five
Q3. Duration of pain before correct diagnosis	1. Just the acute phase, some days . 2. Up to one month . 3. Up to three months. 4. Up to one year. 5. More than one year. 6. Therapy has not been successful so far
Q4. Types of therapy proposed	• Behavioural suggestions • Physical therapy • Oral appliance • Medication • Minor surgery—arthrocentesis • Major surgery • Orthodontics to correct dental occlusion • Prosthodontics to correct dental occlusion • Other strategies to correct dental occlusion
Q5. Perceptions of dental occlusion or mandibular position	0. No. 1. Yes
Q6. Source of specialist referral	1. Referral from general dentist. 2. Online search for expert/specialist. 3. Online patient forums. 4. Referral from another patient

Categorical variables were summarised using frequency distributions, while continuous variables were described using measures of central tendency (mean and median). Additionally, data were expressed as percentages of the total number of participants to provide a clear understanding of the distribution of responses. When possible, the Spearman correlation test was performed to assess correlation levels between variables. Additionally, participants were then divided into two groups according to one variable:

1. patients who have been told by a healthcare provider that TMD problems are due to abnormalities of dental occlusion or mandibular position,
2. patients who have not been told by a healthcare provider that TMD problems are due to abnormalities of dental occlusion or mandibular position.

The Mann–Whitney *U* test was used to determine whether there is a statistically significant difference between the groups for the following variables: number of professionals seen before receiving the diagnosis, amount of time during which patients have been in pain, number of therapies received. The level of significance was set at *p* < 0.05.

## 3 Results

In total, 153 patients (mean age 38.8 ± 14.9 years) answered the survey, of whom 77% were females and 23% were males. Participants were from various countries, with the majority being from Italy (*N* = 115, 75.2%), followed by Romania (*N* = 16, 10.5%). Other countries had fewer representatives (Table 2).

**TABLE 2:** Country of residence of the participants.

Country	
Italy	115
Romania	16
Philippines	3
United Kingdom	3
USA	3
Portugal	1
Finland	1
Ecuador	1
Vietnam	1
Greece	1
Ireland	1
Switzerland	1
Poland	1
Scotland	1
Slovakia	1
Serbia	1
France	1
Uruguay	1

Patients often consulted multiple professionals before receiving a definitive TMD diagnosis. A high number of patients consulted two or three professionals (*N* = 48, 31.4%), while some of them saw up to four or five (*N* = 27, 17.6%). Some patients were diagnosed after the first consultation either by their general dentist (*N* = 25, 16.3%) or an orofacial pain expert/specialist (*N* = 23, 15%). A notable number of patients (*N* = 14, 9.2%) reported not receiving a diagnosis despite consulting multiple professionals (Table 3).

**TABLE 3:** Number of professionals consulted by the patients before receiving the diagnosis—amount of time during which patients experienced pain before receiving correct diagnosis.

Category	Responses options	Frequency (*n*)
Number of professional consulted	I have not received a diagnosis yet, I still have a problem	14
	More than five	16
	Four or five	27
	Two or three	48
	One, just my general dentist	25
	One, but a specialist other than my general dentist	23
Duration of pain before diagnosis	Therapy has not been successful so far	30
	More than 1 year	43
	Up to 1 year	30
	Up to 3 months	15
	Up to 1 month	17
	Some days	11
	Other	7

Patients experienced a wide range of pain duration time before receiving a diagnosis. Almost one‐third of patients experienced pain for more than one year (*N* = 43, 28.1%), and a substantial number never received appropriate therapy (*N* = 30, 19.6%). Some patients experienced pain for up to one year (*N* = 30, 19.6%), up to one month (*N* = 17, 11.1%) and up to three months (*N* = 15, 9.8%) before being correctly diagnosed (Table 3).

The most common treatment proposed was the use of oral appliances (*N* = 81), followed by behavioural suggestions (*N* = 69) and orthodontics (*N* = 40). Other treatments included medications (*N* = 29), minor surgery (*N* = 23), prosthodontics (*N* = 18), and strategies to correct dental occlusion (*N* = 10). Few patients reported being proposed major surgery (*N* = 7) or other therapies (*N* = 8) (Table 4). Table 4 indicated also the number of therapies that have been proposed to the patients, ranging from no therapy up to eight different therapies. Almost half of the patients (*N* = 71) received the proposal of only one type of therapy.

**TABLE 4:** Type and number of therapies that have been proposed to the patients.

Category	Responses options	Frequency (*n*)
Type of therapies proposed	Oral appliance	81
	Behavioural suggestions	69
	Orthodontics	40
	Medication	29
	Minor surgery—arthrocentesis	23
	Prosthodontics	18
	Strategies to correct dental occlusion	10
	Major surgery	7
	Others	8
Number of therapies proposed	No therapy proposed	2
	One type	71
	Two types	38
	Three types	19
	Four types	13
	Five types	6
	Six types	2
	Seven types	1
	Eight types	1

Regarding the perceived cause of TMDs, 101 patients (66.0%) reported that their healthcare providers suggested a relationship between TMD and abnormalities in dental occlusion or mandibular position, whereas 52 providers (34.0%) did not make this connection.

Almost half of the patients found their specialist through a referral from a general dentist (*N* = 72, 47.1%). Other common methods included online searches for specialists (*N* = 36, 23.5%) and recommendations from other patients (*N* = 18, 11.8%). Less common methods included online patient forums, referrals from other doctors and personal networks (Table 5).

**TABLE 5:** Methods adopted by patients to find a specialist.

How did you find the specialist(s) who treated you for TMD?	
Referral from general dentist	72
Online search for expert/specialist	36
Recommendation from another patient	18
Online patient forum	6
Referral from another doctor	7
Others	14

The results of the Spearman correlation showed a moderate positive correlation between the number of professionals visited and the duration of symptoms (*r* = 0.33, *p* < 0.0001). The correlation between the number of therapies received and the number of professionals visited was moderate and statistically significant (*r* = 0.32, *p* < 0.001). The correlation between the number of therapies received and the amount of time for pain duration was as well‐moderate and statistically significant (*r* = 0.30, *p* = 0.0014) (Table 6).

**TABLE 6:** Results of the Spearman association among the different variables.

Spearman associations between variables	Coefficient (*r*)	*p*
Number of professionals visited AND pain duration	0.33	0.0000268
Number of therapies received AND number of professionals visited	0.32	0.0006877
Number of therapies received AND duration of pain	0.30	0.0014

The Mann–Whitney *U* test results that compared the patients who have never been told that their TMD is due to abnormalities of dental occlusion or mandibular position (Group 1) (G1) (*N* = 52) with the patients that have been told by at least one healthcare provider that TMD is related to occlusal features and mandibular position (Group 2) (G2) (*N* = 101) are shown in Table 7. The test showed a significant difference in the number of professionals consulted before receiving the diagnosis (*p* = 0.0062). When the hypothesis that G1 < G2 was set, it resulted that the average number of professionals visited by G1 was significantly lower than G2 (*p* = 0.0032). No statistically significant difference was present between the two groups for the duration of pain (*p* = 0.3) and for the number of therapies that have been proposed (*p* = 0.12).

**TABLE 7:** Mann–Whitney *U* test comparing the differences between the groups that have never been told that TMD is due to abnormalities of dental occlusion or mandibular positions with the patients that have been told by at least one healthcare provider that there is a link between the two variables.

Mann–Whitney *U* test		Number	*p*	*p*
			G1 ≠ G2	G1 < G2
Number of professionals visited before receiving the correct diagnosis	Group 1	52	0.0062	0.0032
	Group 2	101		
Amount of time patients have been in pain before receiving the correct diagnosis	Group 1	52	0.3	
	Group 2	101		
Number of proposed therapies	Group 1	52	0.12	
	Group 2	101		

Abbreviations: G1, Group 1; G2, Group 2.

## 4 Discussion

It is well‐known among orofacial pain specialists that individuals with TMDs represent a vulnerable category of patients [24, 25, 26]. These patients usually come to the attention of the OFP experts after consulting many other practitioners. Since dentists are usually the health practitioners who intercept these patients first, there is the potential risk for those individuals of being exposed to old‐fashioned mechanistic theories focused on what dentists believe (i.e., fixing purported abnormalities in dental occlusion). Indeed, that is what happened with about two‐third of the 153 patients who responded to this survey.

The reason why such patients further look for a second opinion is almost always due to the persistence of pain and dysfunction. The search for the right specialist is further complicated by the contrasting information that patients can find on the internet, as more than half of web pages contain misleading information about TMDs [27]. In addition to that, patients may also encounter some non‐dental healthcare providers who promote themselves as TMD experts, potentially recommending useless or harmful therapies [28]. Under this scenario, orofacial pain specialists often will see patients who were either misdiagnosed or received inappropriate therapies that, in some cases, had irreversible effects [25]. In other words, in many cases the clinical scenario may be more complicated by the previous negative experiences of the TMD patients than by the presence of the TMJ disorder itself [24]. Additionally, it is important to consider the economic and psychological burden that patients must endure before receiving the correct diagnosis. Therefore, it is understandable that some patients inevitably lose their trust in the whole dental profession and are reluctant to initiate a new treatment with a new professional, thereby further delaying the delivery of appropriate care.

These findings suggest that previous experiences of TMD patients may represent the biggest challenge for the subsequent caregivers. Within these premises, the aim of this study was to investigate more in detail, among a random sample of TMD patients, their pathway to find the proper specialist and receive the appropriate treatment. The results confirm that there is still a large number of TMD patients who struggle to find the diagnosis and treatment for their problem. More than a quarter of them (28.1%) had to carry on with their symptomatology for more than one year, while 19.6% had not received an appropriate treatment plan yet. Moreover, patients who were exposed to old gnathological dogmas regarding possible links between temporomandibular disorders, mandible position, and occlusion had to visit a significantly higher number of practitioners before receiving diagnosis.

Such results are in line with other investigations performed on TMD patients. It is a common problem for them to struggle to find appropriate care and to be in a continuous search for a knowledgeable doctor. Their frustration becomes so important that in some cases they even abandon the idea of finding a specialist who can treat them [29]. It has been shown that such a never‐ending search has both an important economic and biological burden, since some TMD patients reported that the treatment plan received by their dentist was to keep drilling teeth in a search for the ‘perfect occlusion’ that could relieve TMD symptoms [30]. Moreover, a recent study shows how practitioners' misconceptions can have profound consequences on patients' beliefs, with detrimental effects on the treatment and prognosis of their TMDs [31].

The present study confirms the need to improve knowledge among professionals as well. Dentists are often the first professionals who screen TMD patients, so they should either provide appropriate modern TMD therapies or send them to the proper specialists. Indeed, another important finding of the present study is represented by the continuing popularity of mechanistic treatments proposed to TMD patients to relieve their symptoms. Around 40 practitioners proposed orthodontic treatment, despite scientific evidence clearly showing the neutrality of orthodontics towards TMDs [21, 32, 33]. Such findings are not fully surprising, considering the belief that still permeates some clinical orthodontic communities about its role as a treatment (or cause) for TMDs [34, 35]. The confusion on the role that occlusal modification can have on TMDs is not uncommon among prosthodontists as well, as shown by a recent survey unveiling the heterogeneous opinions they can have on the topic [36, 37, 38].

The question of why there is so much confusion on TMDs among practitioners may arise, and in the light of the available scientific literature, there could be several possible explanations. In the first instance, the universities should serve as the repository of good practice teaching, but they sometimes foster confusion on TMDs instead. Several papers have shown how dental schools' programs completely lack or dedicate very few numbers of hours to teaching students how to diagnose, treat and manage TMD patients. Moreover, such scenarios are common in different parts of the world, and there are no apparent differences between various continents [39, 40, 41, 42, 43]. In the United States, even at the predoctoral level, only 57% of dental schools teach their students about the importance of the psychological history of the patients, while 58% of them still teach students to perform occlusal evaluation as part of TMD assessment [44]. In China, about 75% of postgraduate students and 85% of dentists claimed that they had never participated in a TMD training programme [44], while in Brazil, only 38.5% of dental schools had an orofacial pain specialist in their faculty team [39]. On the other hand, dental school students who had theoretical and practical experience in TMD and orofacial pain in their training programs meet satisfactory clinical competence [45]. Another source of misinformation arises from the lack of uniformity among postgraduate courses in TMD and orofacial pain, which complicates the possibility to identify the right educational pathway [46, 47, 48]. Finally, a third reason that could explain the widespread lack of knowledge is that the overtreatment of TMD patients based on old gnathological dogmas, such as the pursuit of specific functional occlusal relationships, canine guidance and condylar position, might have a placebo effect and relieve symptoms [49]. Such ‘overtreatment successes’ might be an obstacle that prevents clinicians from altering their knowledge of TMD. Moreover, considering that treatment plans such as orthodontics or prosthodontics rehabilitation provide a higher profit compared to conservative treatment modalities, clinicians could fail to adopt the biopsychosocial model for TMD treatment due to potential conflicts of interest [50]. The ethical push to adopt standard of reference approaches should thus be reinforced [51].

The present study has some limitations, mainly represented by the recruitment modality of TMD patients via an online survey. Such an approach implies a possible risk of selection bias due to the possible higher responsiveness to the questionnaire of TMD patients that had negative clinical experiences. Moreover, the patient's medical history was not collected through the anamnesis but via a series of pre‐selected questions, which introduces a possible memory bias as to what they were told concerning their diagnosis, prognosis and treatment requirements. However, this method allowed for collecting data on a larger sample compared to previous investigations on the same topic [29, 30, 31] and on patients from different geographical areas. Moreover, no clinical investigation was performed, which could be added to future investigations exploring this topic. Another limitation is represented by the cross‐sectional design of the study and the subsequent lack of follow‐up. Based on that, future studies should be performed on patients who have already received the diagnosis of TMD and profile them according to their actual physical and psychological aspects.

## 5 Conclusions

The present study shows that, despite the plea for conservative care by the scientific community and the positive response of most patients, it is not unusual that TMD patients have negative experiences even in terms of delayed diagnosis. Moreover, it results that among dental practitioners there is still confusion on the standard of care for TMD diagnosis and treatment. There is a need to better implement knowledge on temporomandibular disorders among dentists, starting from improving the quality of education at university course level.

## Author Contributions

A.M. contributed to the collection of the data, data processing and writing of the original draft. O.I.S. took part in data processing, writing and editing of the original draft. C.S.G. took part in the editing of the original draft. M.F. contributed to data collection and supervised the investigation. D.M. has been involved in data collection, conceptualisation, methodology, supervision, reviewing and editing of the original draft.

## Consent

All individuals gave their informed consent in accordance with the Helsinki Declaration and understood that they were free to withdraw from the study at any time. The research protocol was approved by the Institutional Review Board of the Orofacial Pain Unit, University of Siena, Siena, Italy (#2023‐0137).

## Conflicts of Interest

The authors declare no conflicts of interest.

## Peer Review

The peer review history for this article is available at https://www.webofscience.com/api/gateway/wos/peer‐review/10.1111/joor.13993.

## Data Availability

The data that support the findings of this study are available on request from the corresponding author. The data are not publicly available due to privacy or ethical restrictions.
